# Decreased renal AT_1_ receptor binding in rats after subtotal nephrectomy: PET study with [^18^F]FPyKYNE-losartan

**DOI:** 10.1186/s13550-016-0209-4

**Published:** 2016-06-23

**Authors:** Basma Ismail, Robert A. deKemp, Tayebeh Hadizad, Kumiko Mackasey, Rob S. Beanlands, Jean N. DaSilva

**Affiliations:** National Cardiac PET Centre, University of Ottawa Heart Institute, 40 Ruskin St., Ottawa, ON K1Y 4W7 Canada; Department of Cellular and Molecular Medicine, University of Ottawa, 451 Smyth Road, Ottawa, ON K1H 8M5 Canada; Department of Radiology, Radio-Oncology and Nuclear Medicine, University of Montreal, University of Montreal Hospital Research Centre (CRCHUM), 900 Rue Saint-Denis, Montréal, Québec H2X 0A9 Canada

**Keywords:** Hypertension, Angiotensin II, PET imaging, ^18^F-losartan, Chronic kidney disease

## Abstract

**Background:**

Significant renal mass reduction induced by 5/6 subtotal nephrectomy (Nx) is associated with a chain of events that culminates in hypertension and chronic kidney disease (CKD). Numerous studies have provided evidence for the role of angiotensin (Ang) II type 1 receptor (AT_1_R) in the promotion and progression of the disease; however, conflicting results were reported on intrarenal AT_1_R levels in CKD models.

**Methods:**

Male Sprague-Dawley rats (*n* = 26) underwent Nx or sham operations. Animals were scanned at 8–10 weeks post-surgery with PET using the novel AT_1_R radioligand [^18^F]FPyKYNE-losartan. Radioligand binding was quantified by kidney-to-blood ratio (KBR), standard uptake value (SUV), and distribution volume (DV). After sacrifice, plasma and kidney Ang II levels were measured. Western blot and ^125^I-[Sar^1^, Ile^8^]Ang II autoradiography were performed to assess AT_1_R expression.

**Results:**

At 8–10 weeks post-surgery, Nx rats developed hypertension, elevated plasma creatinine levels, left ventricle hypertrophy, increased myocardial blood flow (MBF), and reduced Ang II levels compared to shams. PET measurements displayed significant decrease in KBR (29 %), SUV (24 %), and DV (22 %) induced by Nx (*p* < 0.05), and these findings were confirmed by in vitro assays.

**Conclusions:**

Reduced renal AT_1_Rs in hypertensive rats measured with [^18^F]FPyKYNE-losartan PET at 8–10 weeks following Nx support further use of this non-invasive approach in longitudinal studies to better understand the AT_1_R role in CKD progression.

## Background

Significant renal mass reduction induced by subtotal 5/6 nephrectomy (Nx) is a model of chronic kidney disease (CKD), which involves deterioration of renal function due to loss of substantial number of nephrons and compensatory hypertrophy in the remnant kidney. In this model, the development of hypertension, proteinuria, and progressive renal fibrosis eventually leads to end-stage renal disease [1–3]. The incidence of cardiovascular events in CKD is more frequent and severe compared to the normal population [4, 5]. Cardiovascular complications including heart failure or ischemic heart disease represent the leading cause of death in CKD patients [5, 6]. Amongst others, components of the renin angiotensin (Ang) system (RAS), particularly Ang II type 1 receptor (AT_1_R), are known to contribute towards the detrimental effects on the hemodynamic and inflammatory events associated with the pathogenesis of the disease [7–9]. The efficacy of anti-RAS therapies, such as AT_1_R blockers (ARBs) and Ang converting enzyme inhibitors (ACEIs), in slowing progression of renal dysfunction, treating hypertension, and reducing cardiovascular complications confirms the important role of the AT_1_R signaling pathway in these disorders [3, 10, 11]. Numerous large clinical trials demonstrated the beneficial effects of RAS blockade on clinical symptoms and outcomes of patients with CKD; however, marked interindividual variability were detected in response to these treatments ranging from recognizable clinical benefit to non-detectable benefits or even serious adverse effects [12, 13]. PET imaging of AT_1_R may help to predict the response to anti-RAS therapy and personalize medicine.

Many organs contain components involved in the synthesis and actions of the RAS, which are largely independent of the RAS systemic components and not accessible to routine laboratory testing [14, 15]. The presence of a distinct intrarenal RAS adds complexity in interpreting its role in the progression of CKD. In rodents, there are two isoforms of AT_1_R identified in rodents: the AT_1A_R and AT_1B_R [16, 17]. The AT_1A_R represents the homologous form of the human AT_1_R and is widely distributed on luminal membranes throughout the nephron segments [18].

Using different methodologies, previous reports have presented contradictory results on the temporal role played by AT_1_Rs in the progression of kidney diseases. The exact alterations in renal AT_1_Rs are not entirely understood, with studies reporting decrease in cortex and medulla AT_1_R messenger RNA (mRNA) levels in (30–40 %); [19] no change; [20] or increase (>70 %) [9] in the receptor protein expression associated with CKD rodent models.

Non-invasive in vivo imaging of the AT_1_R will allow for the identification of receptor expression abnormalities in CKD, will allow better understanding of the contribution of AT_1_R to the development of the disease, and will aid to guide medical therapies for effective management of patients. [^18^F]FPyKYNE-losartan is a novel PET imaging agent displaying high tissue uptake in the kidney cortex and outer medulla and binding selectivity for AT_1_R over AT_2_R [21]. It binds with high affinity to renal AT_1_Rs (*K*_D_ of 49.4 nM) and has antagonistic efficacy with fourfold less potency reduction of blood pressure (ED_50_ of 25.5 mg/kg) relative to losartan. [^18^F]FPyKYNE-losartan PET imaging exhibited excellent reproducibility in pigs [22]. The current work aims to evaluate the capability of using [^18^F]FPyKYNE-losartan PET to study in vivo renal AT_1_R changes in Nx animal model of CKD.

## Methods

### Animal model

All animal experiments were conducted in accordance with the guidelines of the Canadian Council on Animal Care and with approval of the University of Ottawa Animal Care Ethics Committee. Male Sprague-Dawley rats (*N* = 26; 200–250 g; Charles River Laboratories, Montreal, Canada) were housed in pairs on a 12:12 h light/dark cycle and fed standard rat chow and water ad libitum. Rats were subjected to either sham or Nx surgery in two sittings under total anesthesia with 2 % isoflurane. In the first step, the right kidney was exposed through a lateral dorsal incision, then decapsulated and excised. One week later, the left kidney was exposed in the same way and reduced to 1/3 of its original size by resecting the superior and inferior poles to induce a total of 5/6 Nx [8, 9]. Post-operative analgesia was provided by subcutaneous administration of buprenorphine twice daily for 3 days following surgery. Sham animals underwent the same two surgeries 1 week apart to simulate Nx conditions without removing the kidneys. Animals were weighed at baseline and end of experiment. After sacrifice by decapitation, kidney, heart, and left ventricle (LV) weights were obtained.

### Hemodynamic and biochemical parameters

*Systolic blood pressure* was measured in conscious rats at 8–10 weeks post-surgery using indirect tail-cuff plethysmography (CODA-S2 multi-channel, Kent Scientific). Animals were placed in rat holders and trained for 3 days to the measuring conditions. On the fourth day, six consecutive measurements were recorded from each rat per session, and an average blood pressure was recorded.

*Echocardiography* was carried out under light anesthesia using the Vevo 770 system (VisualSonics) and a 23.5 MHz probe. All studies were performed and analyzed by a single operator. Parasternal long-axis views were recorded as sequential ECG-gated M-mode sweeps (EKV-mode) to generate two-dimensional cines of the left ventricle. Analysis of the results was completed with the VisualSonics cardiac measurements program, and LV wall mass and percent ejection fraction (%EF) were calculated.

*Blood and urine measurements* for creatinine and albumin were assessed at 8–10 weeks post-surgery. Blood samples were collected, centrifuged (4000 rpm, 4 °C) for 10 min, then plasma was stored at −80 °C. Prior to measuring creatinine, plasma proteins were precipitated using deproteinizing sample preparation kit (Biovision). Plasma creatinine levels were determined using a creatinine assay kit (Cell Biolabs) following the manufacturer’s instructions. Urine albumin (corrected to creatinine concentration in urine) was determined. Albumin in urine samples was quantified with a commercially available rat ELISA kit (Genway Biotech).

### Angiotensin II plasma and tissue levels

After decapitation of animals, trunk blood was collected in EDTA tubes containing a cocktail of protease inhibitors, then centrifuged at 4000 rpm for 5 min to obtain plasma. Kidneys were rapidly collected and snap frozen on dry ice. Tissues were homogenized in an acidic ethanol (80 % vol/vol 0.1 N HCl) solution consisting peptidase inhibitors described before [23]. Plasma and tissue samples were stored at −80 °C until the day of assay. Ang II analyses were performed by the Hypertension Core Laboratory at Wake Forest University Health Science Center [24]. Samples were Sep-Pak extracted and measured by RIA (ALPCo, Windham, NH, USA).

### In vivo imaging

Animals were anesthetized (2 % isoflurane) and kept unconscious throughout the scan using the Inveon DPET camera (Siemens Preclinical Imaging). Rats were set for imaging with the microPET scanner as described previously [21]. Images were analyzed with Inveon® Research Workplace software version 1.4 (Siemens Preclinical Imaging) unless indicated otherwise. Dynamic PET images were reconstructed using vendor-provided three-dimensional ordered subset expectation maximization/maximum a posteriori algorithm OSEM3D/MAP (*β* = 1, OSEM3D iterations = 2, MAP iterations = 18) with all corrections enabled. Volumes of interest (VOIs) were defined on reconstructed images to obtain time-activity-curves (TACs) in units of Bq/cc.

### [^13^N]Ammonia blood flow

Heart and kidney perfusion studies were assessed within the same week of [^18^F]FPyKYNE-losartan PET scans. Animals were injected [^13^N]ammonia 55-110 MBq intravenously and scanned for 30 min. Myocardial blood flow (MBF) was quantified using FlowQuant© software [25]. Blood and kidney TACs were generated and flow values (ml/g/min) were produced. For calculation of renal blood flow (RBF), the initial 2 min of the dynamic PET data was used to avoid contamination by plasma metabolites of N-13 radioactivity. The images were reconstructed into 12 × 10 s frames applying the corrections for dead-time, isotope decay, detector efficiencies, and random events. Renal TACs were derived from VOI drawn over renal cortex, and arterial input function was derived from the left atrium (LA). The one-tissue-compartment kinetic model was used to calculate K_1_ for RBF analysis.

### [^18^F]FPyKYNE-Losartan PET

Rats were injected with 18–81 MBq of [^18^F]FPYKYNE-losartan (<1 ml volume) via a 26-gauge catheter into the lateral tail vein. The specific activity ranged from 6.4 to 168.4 GBq/μmol (172—4551 mCi/μmol) at the time of injection, which is similar to the values used previously demonstrating excellent reproducibility in AT_1_R-rich kidney [22]. A dynamic 60-min scan was acquired as 12 × 10 s, 3 × 60 s, and 11 × 300 s frames. The arterial input function was obtained from the average blood pool activities within the LA and LV cavities. Briefly, a sphere was drawn inside the cavity of LA or LV at an early frame (10–40 s post-injection), and an 80 % threshold was used to define the contour of the arterial blood VOI. Kidney VOIs were drawn at frame 16 or 17 (5–15 min post-injection where the highest tissue-to-background contrast was observed) by tracing a segment of the cortex at the inferolateral side of the left kidney away from liver and bowel to avoid spillover, and the final VOI was defined using a 50 % threshold contour. [^18^F]FPyKYNE-losartan retention was measured using kidney-to-blood activity ratio (KBR) and standard uptake value (SUV) and was correlated with Logan-derived distribution volume (DV) values. SUV allowed relative comparison between subjects by normalization to the injected activity and the body weight of the rat. Kidney KBR and SUV values were evaluated at 12.5 min post-injection, which was the time-point displaying maximal tissue-to-blood contrast.

The tracer DV provides a quantifiable parameter for repeated measurements and assessment of repeatability and reliability. Provided the tracer binds to its receptor reversibly, Logan graphical analysis [26], of the PET time-activity data can be used to calculate the DV. In essence, tracer uptake in the kidney is plotted against concentration in the plasma at equilibrium (steady-state). Plotting 0∫^T^C_PET_(t)dt/C_PET_(T)(min) against 0∫^T^C_P_(t)dt/C_PET_(T)(min), where C_PET_(T) is the concentration of tracer in the tissue and C_P_(t) is the concentration of tracer in the plasma, will transform the tissue activity to a linear plot, as if the tracer was injected as a continuous infusion. The slope of this line during the steady-state phase corresponds to an estimate of the DV (ml/cm^3^).

### Western blot

A subset of animals was dedicated to determine tissue AT_1_R protein expression in the left kidney cortex using the Bio-Rad Western blot system following previously described methods [27, 28]. Briefly, rats were sacrificed without anesthesia, and the kidneys were rapidly excised and flash-frozen in liquid nitrogen and powdered and stored at −80 °C until the time of experiment. The tissue was prepared and protein quantification was determined using the BCA protein assay. Protein samples were loaded and separated using 10 % SDS polyacrylamide gel electrophoresis followed by transfer to PVDF membranes. The primary AT_1_R rabbit polyclonal antibody was purchased from Alomone. The membranes were incubated with the primary antibody (1:2000 for 3 h in 2.5 % skimmed milk), washed (5 min × 6 times) and incubated with the secondary antibody (1:5000) for 1 h then washed. Protein bands were visualized using ECL and FlourChem 9900 imaging system. GAPDH was used as a loading control, using mouse primary monoclonal antibody (1:5000) and donkey secondary anti-mouse antibody (1:2000). Data were expressed as integrated density volume (IDV) normalized to GAPDH.

### In vitro autoradiography

In vitro ^125^I-[Sar^1^, Ile^8^]Ang II binding was carried out using the method published previously [29]. Briefly, rats were sacrificed 2–3 days after PET imaging studies to allow direct comparisons. Following decapitation, dissected kidneys were quickly immersed in OCT compound (Tissue-Tek), frozen on dry ice, and stored at −80 °C. The kidneys were sectioned in the axial axis into 20-μm-thick slices at −18 °C with a cryostat. Tissue sections were thaw-mounted on glass slides and stored at −80 °C. On the day of the experiment, the slides were pre-washed in assay buffer (150 mM NaCl, 50 mM sodium phosphate dibasic, 1 mM EDTA, 0.1 mM Bacitracin, 0.1 % BSA) for 15 min then incubated with 0.8 nM ^125^I-[Sar^1^, Ile^8^]Ang II (Perkin Elmer)for 90 min at room temperature in the presence of AT_2_R antagonist, PD 123,319 (10 μM) to determine total (non-AT_2_R) binding or with unlabelled Ang II for non-specific binding. Specific binding of ^125^I-[Sar^1^, Ile^8^]Ang II was calculated as total (non-AT_2_R) minus non-specific binding. After incubation, the slides were washed three times (4 °C) sequentially in buffer, deionized water, and buffer and then dried. Sections were exposed to phosphor imaging plates (Kodak Screen-K, Bio-Rad) for 48 h. Phosphor plates were read at a 100-μm resolution (Bio-Rad Molecular Imager FX) and analyzed using Quantity One Software (Bio-Rad, Philadelphia). Quantification was done manually by tracing the whole kidney cortex for the radioactivity density (counts/mm^2^).

### Statistical analysis

All data are presented as mean ± standard deviation. Results were compared using two-tailed *t* test; *p* < 0.05 was considered significant. *N* values for each comparison are given in the figures and tables. Pearson correlation coefficient (*r*) was calculated to determine correlation between SUV and DV binding parameters.

## Results

### Animals

No significant change in body weight was observed between Nx and sham-operated rats at the end of experiment (Table 1). By contrast, a significant increase (*p* < 0.05) was observed in left kidney (30.3 %), whole-heart (42.3 %), and LV myocardium (33.6 %) weights of Nx animals when normalized to body weight and compared to shams (Table 1).

**Table 1:** Organ weights normalized to body weights in sham and Nx rats at 8–10 weeks post-surgery (*n* = 3–4 per group)

	BW (gm)	Ht/BW	LV/BW	LK/BW
Sham	643.25 ± 63	2.52 ± 0.077	1.47 ± 0.083	2.09 ± 2.236
Nx	637.67 ± 8	3.60 ± 0.619*	1.96 ± 0.297*	3.68 ± 0.512*

Data is presented as mean ± S.D.

*Nx* 5/6 nephrectomy, *BW* body weight, *Ht* heart, *LV* left ventricle, *LK* left kidney

*p < 0.05 vs sham animals; two-tailed *t* test

### Hemodynamic and biochemical parameters

At 8–10 weeks post-surgery, Nx rats had significantly higher blood pressure values in comparison to sham-operated (Table 2; *p* < 0.002). At baseline, measured LV mass and %EF were comparable between groups (data not shown). Compared to shams, LV %EF was not affected in Nx rats, whereas a significant increase in LV mass was detected at 8–10 weeks post-surgery (1201 ± 215 vs 874.4 ± 117, respectively, *p* < 0.002). As a functional marker of renal dysfunction, the level of creatinine in plasma was significantly elevated in Nx rats (Table 2; *p* < 0.01). However, the amount of albumin excreted in urine (corrected for creatinine concentration) was comparable in both groups (Table 2). A significant reduction (−69 %) in plasma Ang II levels (Table 2; *p* < 0.05) was observed in Nx rats at 8–10 weeks post-surgery in comparison to shams. No change in kidney levels of Ang II was observed between nephrectomized and sham-operated rats (Table 2).

**Table 2:** Characteristics and hemodynamic data in sham and Nx rats at 8–10 weeks post-surgery (*n* = 4–5 per group)

	SBP (mmHg)	Plasma Cr (mg/dl)	Urine albumin/Cr (ug/mg)	Plasma Ang II (pg/ml)	Kidney Ang II (pg/mg)
Sham	145.7 ± 18	0.67 ± 0.3	28 ± 1.3	36.53 ± 17.8	16.56 ± 10
Nx	188.4 ± 15.6*	1.36 ± 0.2*	33.66 ± 4.9	11.2 ± 3*	10.47 ± 1.9

Data is presented as mean ± S.D.

*Nx* 5/6 nephrectomy, *BW* body weight, *SBP* systolic blood pressure, *Cr* creatinine, *Ang II* angiotensin II

*p < 0.05 vs sham animals; two-tailed *t* test

### PET studies

MBF as measured by [^13^N]ammonia PET was significantly increased (*p* < 0.05) in Nx rats compared to sham-operated (4.1 ± 0.8 vs 2.7 ± 0.7 ml/g/min, respectively, *p* < 0.002). While no difference in RBF values was found between both groups (5.4 ± 0.9 vs 5.2 ± 1.4 ml/g/min for Nx and sham rats, respectively).

**Fig. 1:**
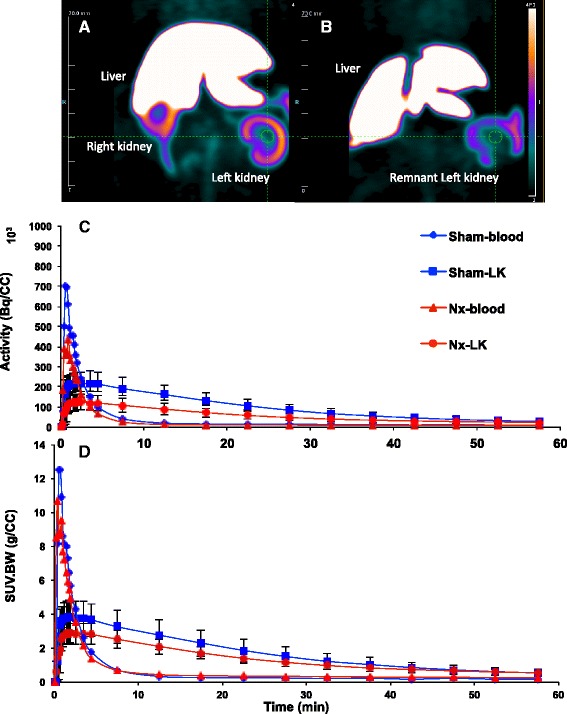
Representative [^18^F]FPyKYNE-losartan coronal view PET images at 5–15 min post-injection showing liver and kidney uptake in **a** sham and **b** Nx rats scanned at 8–10 weeks post-surgery; images are displayed using same SUV scale. Blood and kidney TACs obtained from [^18^F]FPyKYNE-losartan 60 min scans using **c** voxel intensity in Bq/ml and **d** SUV. Data is presented as average ± S.D. *Nx* 5/6 nephrectomy

**Fig. 2:**
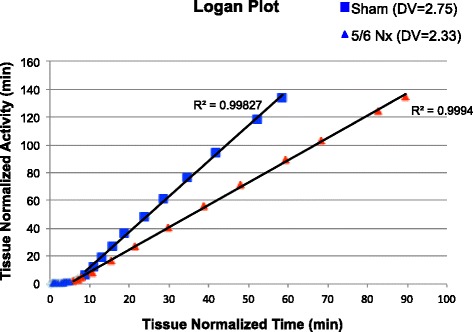
Representative [^18^F]FPyKYNE-losartan Logan plot. The *x-axis* is the adjusted time and the *y-axis* is the adjusted activity. A *straight line* was fitted to each of a sham and Nx rat data. The *slope* of this curve represents the DV value

Representative coronal view PET images are displayed in Fig. 1a for sham and Fig. 1b for Nx at 8–10 weeks post-surgery showing strong liver and kidney signal intensity. Blood and kidney TACs displayed similar pattern for uptake and washout in both groups. However, kidney TACs derived from Nx rats were consistently diminished throughout the 60 min scan by qualitative analysis (Fig. 1c, d). Compared to shams, KBR and kidney SUV obtained from Nx rats were reduced by 29.5 and 24 %, respectively, at 12.5 min post-injection of [^18^F]FPyKYNE-losartan (Table 3). In addition, DV values demonstrated a significant 22 % decrease induced by Nx (Table 3). Logan plots obtained using frames 17–26 (5–60 min) were linear indicating achievement of equilibrium between tissue and plasma (Fig. 2). Correlation between corresponding SUV and DV values was significant (*r* = 0.75).

**Table 3:** [^18^F]FPyKYNE-losartan PET binding parameters in sham and Nx rats at 8–10 weeks post-surgery

	KBR (unitless)	SUV (g/ml)	DV (ml/cm^3^)
Sham	8.0 ± 1.7	2.8 ± 0.9	2.9 ± 0.4
Nx	5.7 ± 1.2*	2.1 ± 0.5*	2.3 ± 0.4*

Data is presented as mean ± S.D.

*Nx* 5/6 nephrectomy, *KBR* kidney-to-blood ratio, *SUV* standardized uptake value, *DV* distribution volume

*p < 0.05 vs sham animals; two-tailed *t* test

### In vitro AT_1_R expression

**Fig. 3:**
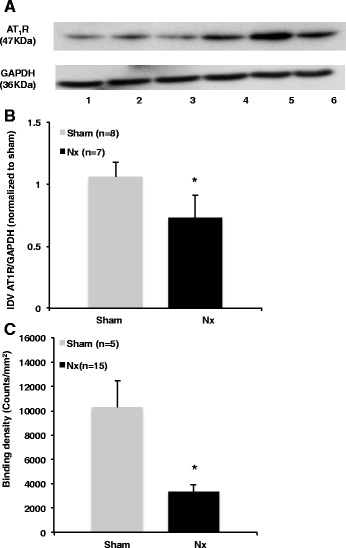
In vitro AT_1_R expression in left kidney cortex at 8–10 weeks post-Nx. Representative Western blot (*lane 1–3*, 5/6 Nx, *lane 4–6*, sham) (**a**); average AT_1_R integrated density value (IDV) normalized to GAPDH and sham (**b**); ^125^I-[Sar^1^, Ile^8^]Ang II binding density (**c**). *p < 0.05 as compared to sham. *N* number of kidney slices

Analysis of left kidney cortex showed a 31 % decrease in AT_1_R protein expression using Western blot technique in Nx animals when compared to shams (*p* < 0.05) at 8–10 weeks post-surgery (Fig. 3a, b), while ^125^I-[Sar^1^, Ile^8^]Ang II binding density displayed a significant decrease of 67 % in the kidneys of Nx rats as compared to shams (*p* < 0.05) (Fig. 3c). The reduction observed in vitro in the AT_1_R renal expression was larger than that measured in vivo by [^18^F]FPyKYNE-losartan PET.

## Discussion

Previous PET studies have explored in vivo renal AT_1_R alterations induced by dietary sodium changes in rats [30] and in a porcine model of renal ischemia [31] but not in the hypertensive Nx rat model of CKD. The removal of one kidney and two thirds of the other in the 5/6 Nx rat model of CKD induces a substantial reduction in the number of functioning nephrons [1, 2]. Subsequently, the remnant kidney size is excessively hypertrophied as a compensatory effect that may start initially as a true adaptive response but becomes maladaptive later in the course of the disease with deterioration of the renal function [3, 19, 32]. In the current study, the presence of renal dysfunction is evident by the development of hypertension and increased plasma creatinine levels, whereas no overt albuminuria is observed in the Nx rats. Early albuminuria is caused mainly by mechanical damage to the glomerular cells leading to impaired selectivity of the glomerular capillary wall and excessive protein ultrafiltration [33], so it may be speculated that this stage of the disease was not reached yet in our experiment. No change in kidney Ang II levels was detected in this study, even though an increase in the remnant kidney was anticipated due to local intrarenal RAS activation [20, 32]. The reduction in plasma Ang II is most likely due to secondary suppression of systemic RAS as reported previously with this model [20, 32].

There is a fair amount of data on the cardiac consequences secondary to renal failure established in this model [9, 34]. Notably, LV hypertrophy is one of the common features reported during the different stages of CKD in animal models and patients [35, 36]. Likewise, LV hypertrophy was observed in the Nx rats in our study. This result is probably attributed to prolonged pressure overload induced by hypertension and not necessarily due to activation of AT_1_R in the heart [4, 5]. It was not possible to visualize [^18^F]FPyKYNE-losartan uptake in the heart due to the very low density of myocardial AT_1_Rs compared to the kidney [37]. Such low cardiac expression requires a very high specific activity of the injected tracer formulation (>7000 Ci/mmol) to prevent saturation of the AT_1_Rs [38].

Cortical RBF was not statistically different in remnant kidney compared to whole left kidney of shams. Previous groups reported an increase in RBF immediately after renal mass reduction that was normalized within 1 week post-surgery [39, 40]. However, normal blood flow can be explained by the presence of intact renal autoregulation in the Nx rat kidneys [9, 41].

The reduction in AT_1_R binding obtained with PET [^18^F]FPyKYNE-losartan in Nx rat kidneys was quantified using different parameters: DVs, SUVs, and KBR values. Physiologically, the Logan-derived DV values are the most appropriate indicators of protein expression and/or receptor-ligand binding potential (Bmax/Kd) for ligands that bind reversibly. However, due to technical limitations, no further corrections were applied for plasma input function, plasma protein binding, and non-specific binding which are implicit to the DV calculation. Hence, to enhance the validity of the measured DV values, PET findings were also represented using semiquantitative analysis (KBR and SUV), where an arterial input function is not indicated. The strong agreement between calculated SUV and DV encourages our confidence in the accuracy of the detected results.

In fact, our imaging data agrees with prior work displaying a decrease in AT_1_R mRNA and protein expression and similar renal mass reduction at different timepoints [19, 42]. Furthermore, Cao et al. reported that the reduction of AT_1_R expression induced an imbalance in the relative proportions of AT_1_ and AT_2_ receptors that is consecutively implicated in progressive renal injury [43]. The lower expression of AT_1_R could be interpreted as a protective mechanism to avoid deleterious effects of hyperactive intrarenal RAS. On the other hand, there are studies that reported opposite effects with elevated AT_1_R expression using Western blot following renal ablation [9, 42]. AT_1_R is implicated in most of the detrimental effects of CKD such as inflammation, renal fibrosis, and renal hypertrophy [9]. A distinct speculation for the inconsistency in the AT_1_R results can be due to the non-specificity of the commonly used AT_1_R antibodies for in vitro assessment as demonstrated in two recent publications [44, 45]. This uncertainty justifies the use of autoradiography in our study in addition to Western assays in assessment of AT_1_R renal expression.

However, to be noted, in vitro autoradiography evaluates only the extent of membrane-bound receptor in non-viable tissue, whereas change in AT_1_R density can be affected by internalization or turnover of the receptor. Activation of AT_1_R signal transduction systems can occur within seconds through G_αq_ and IP_3_ or within minutes to hours through MAP kinase and JAK/STAT systems [46]. Moreover, AT_1_Rs are endocytosed within 10 min after activation, with ~25 % recycled to plasma membrane and the remainder degraded in lysosomes [47]. Consequently, the dynamic nature of AT_1_R is a limitation for the accuracy of in vitro measurements and represents an added value for in vivo PET as an investigative tool to detect total receptor changes.

A non-invasive means for assessing renal AT_1_R signaling at various stages of CKD in vivo would advance our understanding of the receptor abnormalities associated with progression of the disease and therapy response in patients. Measurements of a local tissue RAS component (such as AT_1_Rs) in clinical or experimental CKD studies may be more predictive of the degree of renal injury compared to studies targeting the circulating RAS components. Nevertheless, it is important to note that AT_1_R regulation is cell and tissue specific [48], whereas a major drawback with PET imaging is its poor resolution (especially in rodents) to delineate specific cellular localization of [^18^F]FPyKYNE-losartan accumulation within the kidney.

## Conclusions

The present study is the first to provide in vivo evidence for a reduction of renal AT_1_R cortical expression in a rat model of CKD at 8–10 weeks post-Nx. The robustness of PET findings confirmed by in vitro AT_1_R expression suggests that [^18^F]FPyKYNE-losartan is a promising PET tracer for longitudinal imaging renal AT_1_R in multiple pathological conditions. Such an approach in clinic would present a unique opportunity to advance understanding of the pathophysiology of various diseases including myocardial infarction, hypertension, vascular, and renal failure.

## Abbreviations

ACEIs: Ang converting enzyme inhibitors
Ang: angiotensin
ARBs: AT_1_R blockers
AT_1_R: angiotensin (Ang) II type 1 receptor
CKD: chronic kidney disease
DV: distribution volume
EF: ejection fraction
IDV: integrated density volume
KBR: kidney-to-blood ratio
LA: left atrium
LV: left ventricle
MBF: myocardial blood flow
Nx: 5/6 nephrectomy
RAS: renin angiotensin (Ang) system
RBF: renal blood flow
SBP: systolic blood pressure
SUV: standard uptake value
TAC: time-activity-curve
VOI: volume of interest
